# Host-defense piscidin peptides as antibiotic adjuvants against *Clostridioides difficile*

**DOI:** 10.1371/journal.pone.0295627

**Published:** 2024-01-22

**Authors:** Adenrele Oludiran, Areej Malik, Andriana C. Zourou, Yonghan Wu, Steven P. Gross, Albert Siryapon, Asia Poudel, Kwincy Alleyne, Savion Adams, David S. Courson, Myriam L. Cotten, Erin B. Purcell

**Affiliations:** 1 Department of Chemistry and Biochemistry, Old Dominion University, Norfolk, Virginia, United States of America; 2 Biomedical Sciences Program, Old Dominion University, Norfolk, Virginia, United States of America; 3 Department of Applied Science, William & Mary, Williamsburg, Virginia, United States of America; 4 Irvine Department of Physics and Astronomy, University of California, Los Angeles, California, United States of America; 5 Ivrine Department of Developmental and Cell Biology, University of California, Los Angeles, California, United States of America; University of New South Wales, AUSTRALIA

## Abstract

The spore-forming intestinal pathogen *Clostridioides difficile* causes multidrug resistant infection with a high rate of recurrence after treatment. Piscidins 1 (p1) and 3 (p3), cationic host defense peptides with micromolar cytotoxicity against *C*. *difficile*, sensitize *C*. *difficile* to clinically relevant antibiotics tested at sublethal concentrations. Both peptides bind to Cu^2+^ using an amino terminal copper and nickel binding motif. Here, we investigate the two peptides in the apo and holo states as antibiotic adjuvants against an epidemic strain of *C*. *difficile*. We find that the presence of the peptides leads to lower doses of metronidazole, vancomycin, and fidaxomicin to kill *C*. *difficile*. The activity of metronidazole, which targets DNA, is enhanced by a factor of 32 when combined with p3, previously shown to bind and condense DNA. Conversely, the activity of vancomycin, which acts at bacterial cell walls, is enhanced 64-fold when combined with membrane-active p1-Cu^2+^. As shown through microscopy monitoring the permeabilization of membranes of *C*. *difficile* cells and vesicle mimics of their membranes, the adjuvant effect of p1 and p3 in the apo and holo states is consistent with a mechanism of action where the peptides enable greater antibiotic penetration through the cell membrane to increase their bioavailability. The variations in effects obtained with the different forms of the peptides reveal that while all piscidins generally sensitize *C*. *difficile* to antibiotics, co-treatments can be optimized in accordance with the underlying mechanism of action of the peptides and antibiotics. Overall, this study highlights the potential of antimicrobial peptides as antibiotic adjuvants to increase the lethality of currently approved antibiotic dosages, reducing the risk of incomplete treatments and ensuing drug resistance.

## Introduction

The anaerobic spore-forming intestinal pathogen *Clostridioides difficile* (*C*. *difficile)* infects the mammalian large bowel and releases protein toxins, resulting in diarrheal infections with a profound effect on health care costs and patient quality of life[1,2]. In the United States alone, it is responsible for nearly 500,000 infections, 25,000 deaths, and approximately $6 billion in hospitalization costs per year [1]. One fourth to one third of *C*. *difficile* infection (CDI) patients experience recurrent infection, and each round of failed antibiotic therapy increases the risk of recurrence from 30% after a first infection to 45% and 64%, respectively after the second and third occurrences, in a so-called ‘recurrence elevator’ [3]. On average, an initial CDI diagnosis increases an individual patient’s health care costs by $24,000, while each additional recurrence costs $28,000-$60,000, with the overall burden on the U.S. health care estimated to be $5–6 billion per year [4,5]. Prior exposure to antibiotics such as clindamycin, cephalosporins, and fluoroquinolones that disrupt the commensal gut microbiota is the single largest risk factor for CDI, although many other factors such as proton pump inhibitor usage and advanced age contribute to the risk of infection [6]. While CDI has historically been a health-care associated infection, community acquired CDI is becoming more prevalent [7]. *C*. *difficile* persists in the environment and spreads between patients in the form of metabolically dormant spores, which are impervious to antibiotics that target metabolic or biosynthetic processes [8]. Plasma membranes within spores are highly compressed and less dynamic and permeable to exogenous materials than cell membranes in germinated spores or vegetative cells [8,9].

Until recently, the leading antibiotic treatment for CDI was metronidazole, a broad-spectrum antibiotic that inhibits nucleic acid synthesis [10–12]. However, metronidazole has lost clinical efficacy due to the emergence of resistant *C*. *difficile* strains and as of 2017 is no longer recommended as a front-line therapy against CDI [11,12]. Nevertheless, it is still widely prescribed, possibly due to financial considerations [13]. Vancomycin, which inhibits bacterial cell wall synthesis and is specific to Gram-positive bacteria, is the currently recommended treatment for initial CDI diagnosis [11,12]. Clostridial vancomycin resistance is not currently widespread but has been documented and appears to be increasing as metronidazole usage is replaced by vancomycin, increasing the selective pressure favoring vancomycin resistance [13–16]. Fidaxomicin, a macrolide antibiotic that inhibits transcription initiation by RNA polymerase, treats CDI with lower recurrence rates than vancomycin or metronidazole but is not widely prescribed, likely due to its relatively high cost [12,13,17,18]. Clinical isolates of *C*. *difficile* with reduced fidaxomicin susceptibility have been isolated, suggesting that fidaxomicin-resistant CDI will be medically relevant in the future [16,19].

Currently, the most clinically effective treatment for CDI is the replenishment of the protective gut microbiota through fecal transplants. This procedure has a high risk of introducing other pathogens. Given the risk of this approach for immunocompromised patients, there is heightened interest in developing new strategies to prevent and treat CDI [20]. Alleviating the symptoms of infection by neutralizing the clostridial toxins is one active area of research [21]. Combinatorial treatments exploiting synergy between agents that are independently bacteriostatic or bactericidal are another promising field of research, as bacteria would need to evolve two separate resistance mechanisms to successfully evade such treatments [22,23].

Host-defense peptides (HDPs), found in all classes of life, are small, typically cationic, peptides which are produced as an endogenous defense mechanism against pathogens [24–30]. HDPs kill bacteria by a number of mechanisms, including disruption of cell membrane structural integrity and penetration into the bacterial cytoplasm to attack intracellular targets [22,31–39]. Models of membrane-active HDPs suggest potential mechanisms ranging from horizontal peptide insertion within the lipid phase to the formation of transient transmembrane pores [22,40–42]. HDPs are attractive candidates for synergistic interactions with antibiotics, as they have a non-specific electrostatic affinity for the negatively charged lipid headgroups in bacterial cell membranes, to which it would be difficult for bacteria to evolve specific resistance mechanisms [22]. HDPs with high membrane activity are more likely to exhibit antibiotic synergy, suggesting that they could permeabilize the bacterial cell membrane and increase antibiotic penetrance and bioavailability [43]. The human HDPs LL-37 and HBD3 perturb *C*. *difficile* cell membrane integrity and increase susceptibility to multiple classes of antibiotics, including tetracylines [44–48]. Even some fluoroquinoline and β-lactam antibiotics, which are normally ineffective against *C*. *difficile* and associated with increased CDI susceptibility, exhibit synergistic interactions with HDPs to kill *C*. *difficile* [45,47].

Piscidins are cationic HDPs found in fish that exhibit broad-spectrum antimicrobial activity in anaerobic environments and are bactericidal against *C*. *difficile* in anaerobic environments [42,49–56]. Piscidins are known to target bacterial cell membranes, forming α-helices upon membrane binding and physically inserting themselves into the lipid bilayer and causing oxidative damage to component lipids [42,51,52,57–63]. They have garnered biomedical interest due to the strong potency and favorable therapeutic indexes of naturally-occurring piscidins and some analogs [63–65]. Most piscidins have an amino terminal copper and nickel binding motif that enables them to bind to Cu^2+^ with nanomolar affinity, resulting in nuclease activity against intracellular DNA [52,55,59,66]. Piscidin 1 (p1) is more active against membranes while piscidin 3 (p3) is a more potent nuclease [52,58]. Piscidins are capable of permeabilizing membranes from cells and synthetic lipid vesicles made to model the cell membranes, and this effect is enhanced by metal binding [42,57,58,62,67,68].

We have previously shown that p1 and p3 are cytotoxic against both historical and modern epidemic strains of *C*. *difficile* [53]. Both peptides associate with clostridial cells at sites of membrane curvature and effectively kill *C*. *difficile* at micromolar concentrations [53]. p1 is effective at lower concentrations *in vitro* than p3. p1 inhibits clostridial growth and kills exponentially growing *C*. *difficile* at 4 μM while p3 is active at 8 μM, comparable to the 2–4 μM effective concentrations achieved against *C*. *difficile* by hybrid peptides based on porcine and bee HDPs [53,69]. In anaerobic environments, the apo- and metallated piscidins were equally effective, suggesting that the increased oxidative potential of piscidin-copper complexes compared to the peptides alone depends on atmospheric oxygen [53]. Here, we hypothesized that as piscidins are active at the cell membrane, they could help resuscitate the activity of antibiotics against *C*. *difficile* by facilitating access to their targets. We investigated the co-application of sublethal concentrations of piscidins with antibiotics commonly used to treat CDI and determined that the HDPs can increase the efficacy of medically relevant antibiotics against *C*. *difficile*. To explain this adjuvant effect, we performed mechanistic studies with cells and model membranes, and used antibiotics featuring different targets (e.g., cell wall versus intracellular targets).

## Materials and methods

### Materials, chemicals, bacterial strains, and growth conditions

All materials were purchased from Fisher Scientific unless otherwise indicated. *C*. *difficile* R20291 was cultured in BHI (Brain-Heart Infusion; VWR) medium supplemented with 5% Bacto Yeast Extract (Thermo Fisher Scientific) [70]. *C*. *difficile* was cultured anaerobically at 37°C in a Coy Type B vinyl anaerobic chamber (Coy Laboratory Products, Grass Lake, MI) with an atmosphere of 85% N_2_, 10% CO_2_, 5% H_2_. All plastic consumables were equilibrated in the anaerobic chamber for a minimum of 72 h prior to use. *C*. *difficile* was exposed to ciprofloxacin (Cayman Chemicals), erythromycin (Acros Organics), fidaxomicin (Cayman Chemicals), metronidazole (BTC), vancomycin (VWR), or Cu_2_SO_4_ (MP Biomedicals) at the indicated concentrations. Taurocholic acid (Sigma Life Science) was added to BHIS agar plates at a concentration of 0.1% for spore germination.

### Peptide synthesis

The piscidin peptides p1 and p3 were prepared using previously established protocols [42,52–54,71]. Briefly, the peptides were synthesized at the University of Texas Southwestern Medical Center (Houston, TX, USA) and Bio-Synthesis Inc. (Lewisville, TX, USA). Following synthesis by Fmoc solid phase peptide synthesis and purification (>95%) by reverse phase HPLC, the lyophilized peptides were solubilized in dilute hydrochloric acid to exchange the trifluoroacetate ions for chloride ions. To remove any excess salt, dialysis was carried out. To prepare the samples for biological assays, the dialyzed peptides were dissolved in water and their concentration determined by amino acid analysis performed by the Protein Chemistry Laboratory at Texas A&M University (College Station, TX, USA) or Amino Acid Analysis Service Laboratory (Damascus, OR, USA). Consistent results were obtained when identical stocks were analyzed by both laboratories. To prepare the peptide-Cu^2+^ constructs, a 1:1 stoichiometric amount of CuCl_2_ was added to each peptide. To ensure optimal conditions for metal binding, the pH of the solution, which tends to drop due to the deprotonation of the four nitrogen sites involved in metal coordination, was adjusted to 7.4 using NaOH. The samples were frozen and stored at ‐20°C until needed.

### Growth inhibition assays

Two-fold serial dilutions of BHIS media containing the indicated concentrations of antibiotics were prepared in sterile 96 well plates (ThermoFisher Scientific). Piscidins and piscidin-Cu^2+^ complexes were added at a concentration of 2 μM for p1 and p1-Cu^2+^ and 4 μM for p3 and p3- Cu^2+^, 0.5X the previous determined inhibitory concentrations [53]. Wells were inoculated 1:10 with exponentially growing *C*. *difficile* R20291 cultures (OD_600_ 0.4–0.6). Inoculated plates were incubated anaerobically at 37°C for 16 h and then removed from the chamber. Plate exteriors were sanitized with bleach and culture density was measured at 630 nm in a BioTek Synergy HT plate reader (Winooski, VT). Effective antibiotic concentrations were defined as those sufficient to inhibit overnight growth in the presence of peptides by at least 90% compared to untreated controls containing no peptides or antibiotics. All experiments were performed with a minimum of three samples, and the means and standard deviations were reported. To analyze the effects of the antibiotics, samples containing peptides and antibiotics were compared to samples containing only peptides by two-way analysis of variance using GraphPad Prism 6.

### Enumeration of colony forming units

200 μL samples of BHIS broth containing the indicated concentrations of antibiotics and/or piscidins were inoculated using a 1:10 ratio, in 96 well plates, with exponentially growing *C*. *difficile* R20291 cells and incubated for 16 h at 37°C. Samples were spread as duplicates on BHIS agar plates containing 20 μg/mL erythromycin. These plates were incubated anaerobically at 37°C for 48 h before the viable colonies were counted.

### Spore viability assays

To isolate spores, exponentially growing *C*. *difficile* R20291 was inoculated 1:10 into 50 mL of BHIS broth and grown anaerobically at 37°C for 16 h. This culture was removed from anaerobic chamber and ethanol was added to a final concentration of 50%. The sample was rocked aerobically for 6 h. The absence of viable vegetative cells was confirmed by spreading volumes up to 1 mL on BHIS plates and incubating anaerobically for 48 h. 100 μL samples of R20291 spores in PBS were mixed 1:1 with PBS or with PBS containing p1 or p3. Peptides were tested at final concentrations corresponding to 1X and 2X their previously observed inhibitory concentrations. All the samples were incubated in the chamber at 37°C for 16 hrs. After incubation, 50 μL aliquots of each sample were plated in triplicate on BHIS plates containing 20 μg/mL erythromycin and 0.1% taurocholic acid agar plates to induce germination [72]. An additional 50 μL aliquot was plated on BHIS containing 20 μg/mL erythromycin to ensure that there was no contamination with vegetative cells. The plates were incubated in the chamber at 37°C for 48 h before the viable colonies were counted.

### Propidium iodide uptake assays

4 mL samples of *C*. *difficile* cultures in late exponential growth (OD_600_ 0.7–0.8) were treated anaerobically at 37°C with 20 μg/mL propidium iodide (PI; Biotium) and inhibitory concentrations of piscidins or piscidin-copper complexes (4 μM for p1 and p1Cu, 8 μM for p3 and p3Cu). Samples containing apo-peptides and metalated peptides were incubated for 30 min and 10 min, respectively. After incubation, cells were centrifuged at 704 rfc for 5 minutes and resuspended in 1.5 mL fresh BHIS broth. Negative controls were incubated with PI for 10 or 30 minutes but contained no piscidin. Positive controls were removed from the anaerobic chamber and incubated aerobically for 1 h at room temperature with propidium iodine and 50% ethanol. Within the anaerobic chamber, samples were injected into rose chambers for microscopic imaging as detailed in [53]. The rose chambers were removed from the anaerobic chamber and the outsides were sterilized with 70% ethanol and 10% bleach. Samples were imaged on a Nikon Ti-E inverted microscope equipped with Nikon Perfect Focus System, apochromat TIRF 60X Oil Immersion Objective Lens (numerical aperture 1.49), a condenser (numerical aperture 0.52), pco.edge 4.2 LT sCMOS camera, and SOLA SE II 365 Light Engine as well as complementary DIC components. Stage temperature was maintained at 36.5°C +/− 0.5°C using a Nevtek Air Stream microscope stage warmer and home-built microscope enclosure. Samples were imaged with time-lapse differential interference contrast (DIC) microscopy and propidium iodide was monitored at excitation/emission wavelengths of 535/617 nm.

### Membrane leakage assays

The synthetic lipids 1-palmitoyl-2-oleoyl-sn-glycero-3-phosphoglycerol (POPG) and 1-palmitoyl-2-oleoyl-3-(β-D-glucosyl)-sn-glycerol ((16:1–18:1) DG-glucose) were purchased from Avanti Polar Lipids (Alabaster, AL). POPG and DG-glucose (4 μmol of total lipids) dissolved in chloroform were mixed in a 2:1 molar ratio prior to drying under a flow of nitrogen. After overnight drying under vacuum, the lipid film was hydrated with 300 μL of 80 mM calcein dye and 4 freeze-thaw cycles were carried out. Next, large unilamelar vesicles (LUVs) were made by extruding the lipid mixture through a 0.1 μM polycarbonate membrane filter (Whatman, Florham Park, NJ). To remove the free calcein dye, the LUVs were run through a size exclusion Sephadex G-50 column (GE Healthcare, Pittsburgh, PA). The mobile phase featured HEPES buffer (50 mM HEPES, 100mM NaCl, 3mM EDTA, 0.01% w/v NaN_3_, pH 7.4). The LUVs were then diluted (1:25 dilution) to approximately 30 μM and aliquoted in a 96 well plate (180 μL per well). The precise concentration of POPG, which made up 66% of the total lipid content, was determined by a phosphorus assay [73]. Stock solutions of p1, p1-Cu^2+^, p3, and p3-Cu^2+^ (150 μM) were serially diluted to be at 10X of the concentration needed to reach the following peptide-to-lipid ratios P1 (P1-Cu^2+^)/L = 1:512, 1:256, 1:128, 1:64, 1:32, 1;16, 1:8, and 1:4 and P3/L (P3-Cu^2+^) = 1:256, 1:128, 1:64, 1:32, 1;16, 1:8, 1:4, and 1:2. Each well containing 180 μL of LUVs was exposed to 20 μL of a peptide solution prepared at a given peptide-to-lipid ratio. Each concentration was run in triplicates. After 1 h incubation at either 37°C or 45°C, with shaking conditions of 140 rpm, the fluorescence of the calcein dye was measured in a BioTek H4 Synergy Hybrid Microplate Reader using excitation and emission wavelengths of 490nm and 520nm. The obtained fluorescence readings were normalized using the following equation:

$$\%\;Fraction\;Calcein\;Leakage\;=\frac{I-I_{background}}{I_{Triton}-I_{background}}\times \;100\;(1)$$

where *I* is the fluorescence intensity measured after exposure to the peptide, I_background_ is the fluorescence measured for the negative control (LUVs alone), and I_Triton_ is the fluorescence intensity measured for the positive control (LUVs with 20μL of 1% v/v of the detergent Triton X-100).

The normalized data was fitted using GraphPad Prism to determine the half-maximal effective concentration for each of the peptides as an EC_50_ value against the P/L ratio. The EC_50_ represents the amount of peptide needed to lyse 50% of the vesicles [74].

### Electron microscopy

Overnight cultures were prepared by inoculating individual colonies into 3 mL BHIS broth and grown for 14–16 h at 37°C in the anaerobic chamber. The overnight cultures were diluted 1:20 into 4 mL BHIS broth and the cultures were grown until they reached exponential phase (OD_600_ 0.4–0.6). Inside the chamber, 1 mL of the exponential phase cultures were treated in test tubes for 5 minutes with: 1) no treatment, 2) 0.5 μg/mL vancomycin, 3) 4 μM p1-Cu^2+^, or 4) 4 μM p1-Cu^2+^ and 0.5 μg/mL vancomycin. After treatment, the samples were transferred to 2 mL Eppendorf tubes and centrifuged for 2 minutes to collect the pellets. The pellets were individually suspended in 1 mL of 1X phosphate-buffered saline solution with 4% paraformaldehyde, prepared as described in [75]. The samples were fixed in the anaerobic chamber for 45 min. After fixation, the eppendorf tubes were removed from the chamber and the exteriors were sterilized with SporGon (Thomas Scientific), 10% bleach, and 70% ethanol (VWR). Samples were incubated on 200 mesh formvar-coated copper grids (Electron Microscopy Sciences, Hatfield, PA) that were glow-discharged. Grids were washed with water, incubated with 2% uranyl acetate, washed with water, dried overnight at room temperature, and imaged using a JEM-2100F TEM (JEOL, Akishima, Japan) at a voltage of 200 kV and using a Gatan OneView camera. The percentage of lysed cells in each condition and the standard error of each condition were compared using the Wilson confidence interval.

## Results

### Piscidins synergize with multiple classes of antibiotics

We have previously reported that both p1 and p3 are bactericidal against *C*. *difficile* at 4 μM and 8 μM concentrations, respectively [53]. As HDPs have previously been reported to potentiate ineffective antibiotics against *C*. *difficile*, we hypothesized that they could increase the efficacy of metronidazole, vancomycin, and fidaxomicin [44]. These antibiotics target different cellular components, providing us with a way to identify whether increased efficacy relies on specific mechanistic aspects of the antibiotics and peptides. We tested this by inoculating *C*. *difficile* R20291 into media containing metronidazole, fidaxomicin, and vancomycin in the presence or absence of piscidins at half of the peptide concentration required to inhibit *C*. *difficile* growth overnight [53].

**Fig 1:**
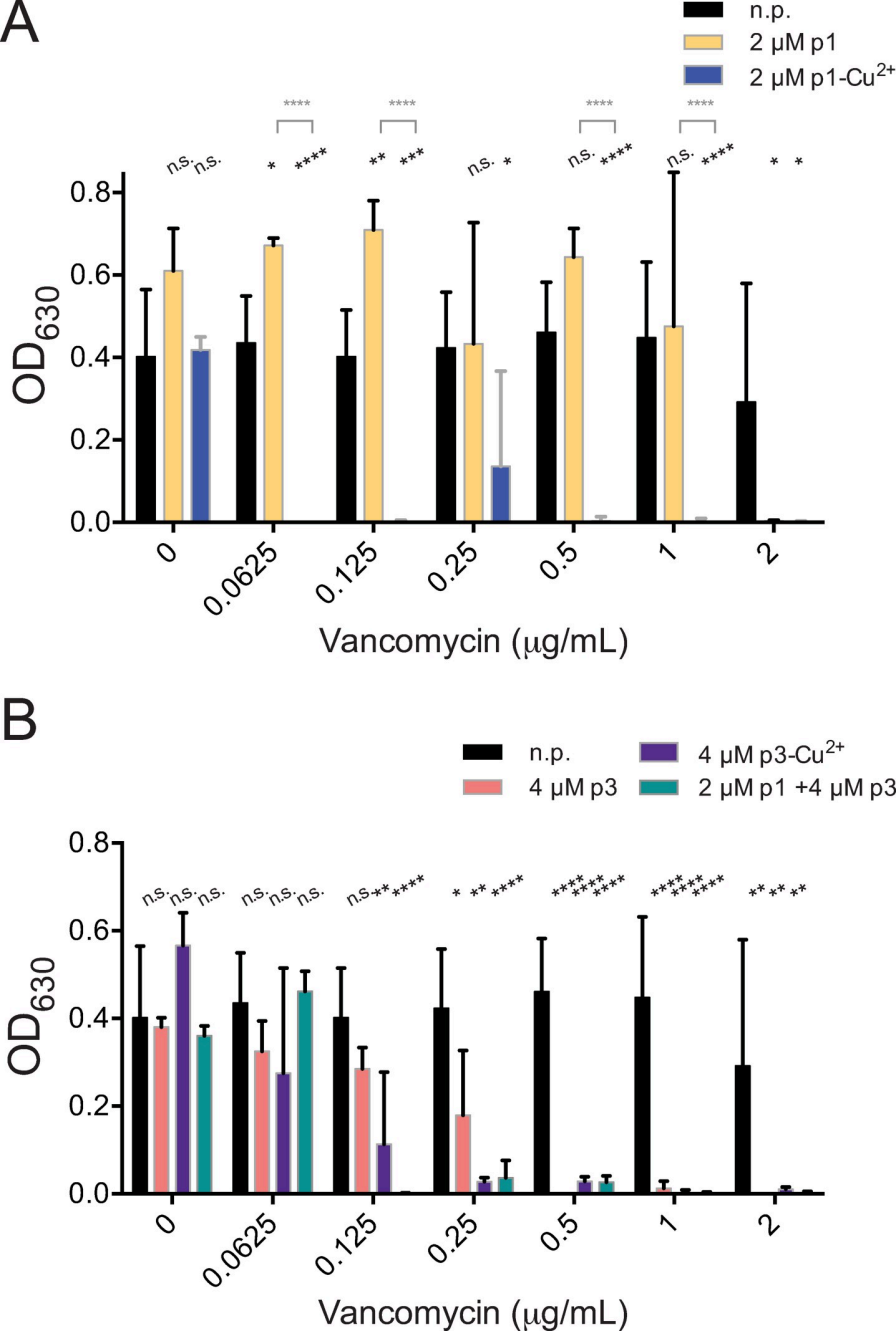
Piscidins synergize with vancomycin. Culture density after overnight growth in the presence of the indicated concentrations of vancomycin and subinhibitory concentrations of (A) p1 or p1-Cu^2+^ or (B) p3 or p3-Cu^2+^complex. Data shown represent the means and standard deviations of at least three samples. Conditions are compared by two-way ANOVA. The comparison between samples containing vancomycin and those containing vancomycin and peptides is shown in black. When comparisons between the apo and holo forms of the peptides are statistically significant, they are shown in grey. n.p. no peptide, n.s. not significant, * *p* > 0.05, ** *p* > 0.01, *** *p* > 0.001, **** *p* > 0.0001.

We first tested the peptides on vancomycin, which targets the cell walls of Gram-positive bacteria, for the ability of vancomycin to reduce overnight growth by at least 90%. We have previously reported that vancomycin inhibits the growth of *C*. *difficile* R20291 at concentrations at or above 4 μg/mL (Table 1) [76]. Using 2 μM p1 improved vancomycin efficacy by a factor of two, reducing its effective concentration from the previously observed 4 μg/mL to 2 μg/mL (Fig 1A and Table 1) [76]. In the case of 2 μM p1-Cu^2+^, the improvement with the peptide was 64-fold, with an effective vancomycin concentration of 0.0625 μg/mL. We note some breakthrough growth in the combinatorial condition containing 0.25 μg/mL vancomycin and 2 μM p1-Cu^2+^. This is due to a single sample out of the set, suggesting contamination or an inoculation error (Fig 1A and Table 1). With 4 μM p3 and p3-Cu^2+^, the effective vancomycin concentrations are decreased 8- and 16-fold to 0.5 and 0.25 μg/mL, respectively (Fig 1B and Table 1). The combination of 2 μM p1 and 4 μM p3 improved the effective vancomycin concentration by a factor of 32 to 0.125 μg/mL (Fig 1A and 1B and Table 1). Overall, all of the peptides tested improved the activity of vancomycin, with p1-Cu^2+^, the more membrane active peptide, most effective at boosting this antibiotic that acts on cell walls.

**Table 1:** Concentrations (μg/mL) of antibiotics necessary to suppress 16-hour growth of *C*. *difficile* in combination with the indicated HDPs.

Antibiotic	No peptide	2 μM p1	2 μM p1Cu	4 μM p3	4 μM p3Cu	2 μM p1 4 μM p3
Vancomycin	4.000	2.000	0.0625	0.500	0.250	0.125
Metronidazole	2.000	0.500	1.0000	0.0625	1.000	0.125
Fidaxomicin	0.256	0.0500	0.0125	0.050	>0.100	0.00625
Ciprofloxacin	128	n/a	n/a	n/a	n/a	2.000

Antibiotic concentrations are given in μg/ml.

**Fig 2:**
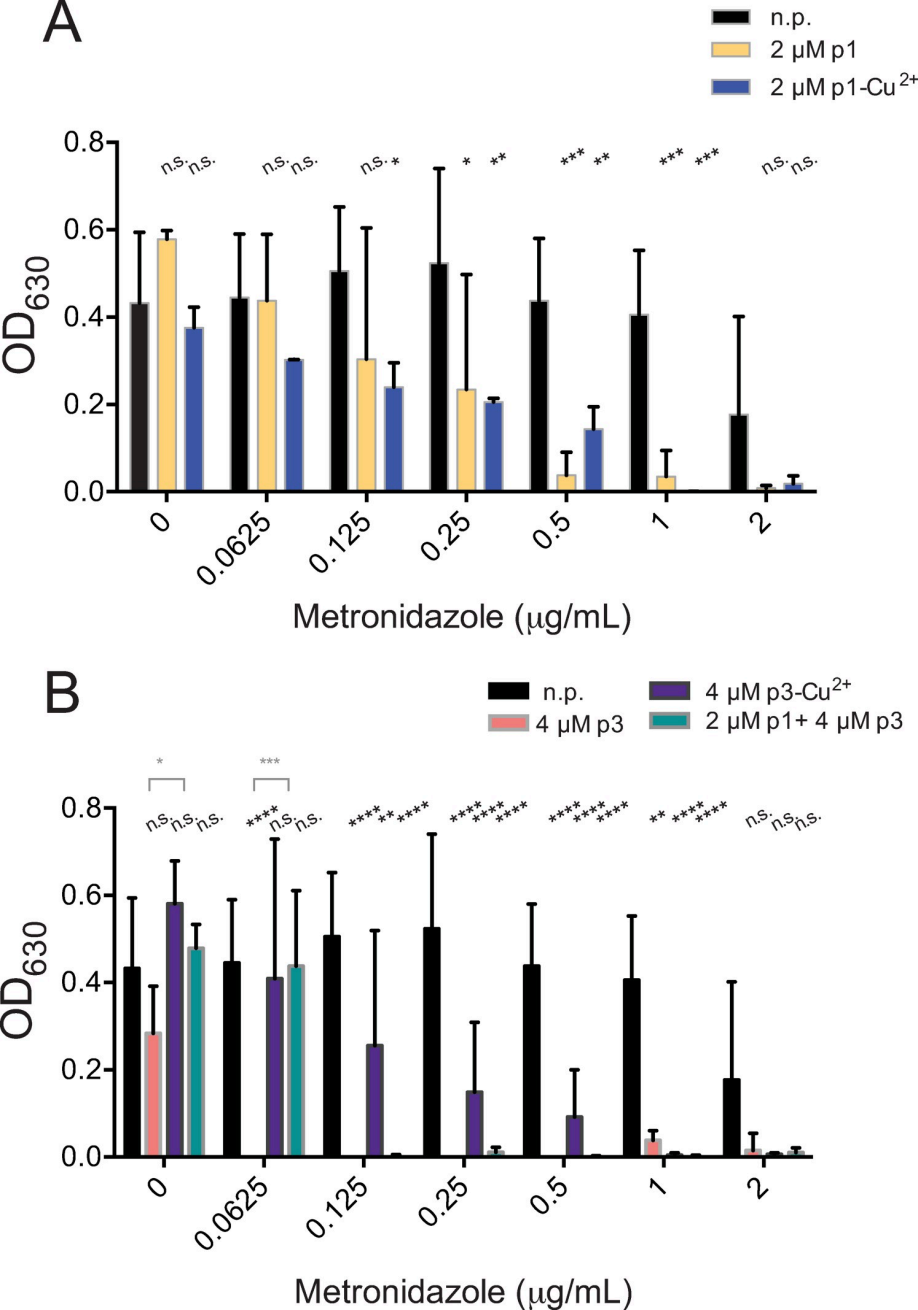
Piscidins synergize with metronidazole. Culture density after overnight growth in the presence of the indicated concentrations of metronidazole and subinhibitory concentrations of (A) p1 or p1-Cu^2+^ or (B) p3 or p3-Cu^2+^. Data shown represent the means and standard deviations of at least three samples. Conditions are compared by two-way ANOVA. The comparison between samples containing metronidazole and those containing metronidazole and peptides is shown in black. When comparisons between the apo and holo forms of the peptides are statistically significant, they are shown in grey. n.p. no peptide, n.s. not significant, * *p* > 0.05, ** *p* > 0.01, *** *p* > 0.001, **** *p* > 0.0001.

Next, we tested antibiotics that have intracellular targets. We started with metronidazole, which targets protein synthesis by binding to DNA [10–12]. We found that 2 μM p1 improved efficacy of metronidazole four-fold, from 2 μg/mL down to 0.5 μg/mL (Fig 2A and Table 1) [10]. Metalation of the peptide did not appear to boost the peptide activity since the effective metronidazole concentration improved only two-fold, decreasing the concentration to 1 μg/mL (Fig 2A and Table 1). Remarkably, 4 μM p3, which condenses DNA very effectively [77], improved the metronidazole efficacy 32-fold with an effective concentration of 0.0625 μg/mL, while p3-Cu^2+^ only reduced the effective concentration two-fold, down to 1 μg/mL (Fig 2B and Table 1). The combination of 2 μM p1 with 4 μM p3 enhanced metronidazole activity 16-fold, decreasing the effective concentration to 0.125 μg/mL. Hence, it was more effective than apo-p1 or either metallated peptide but less effective than p3 (Fig 2A and 2B and Table 1). Overall, all of the peptides improved the efficacy of metronidazole by a factor of at least 2 (Fig 2A and 2B). Importantly, p3 was the most adjuvant to metronidazole with a 32-fold improvement, correlating with the ability of both antimicrobials to target DNA.

**Fig 3:**
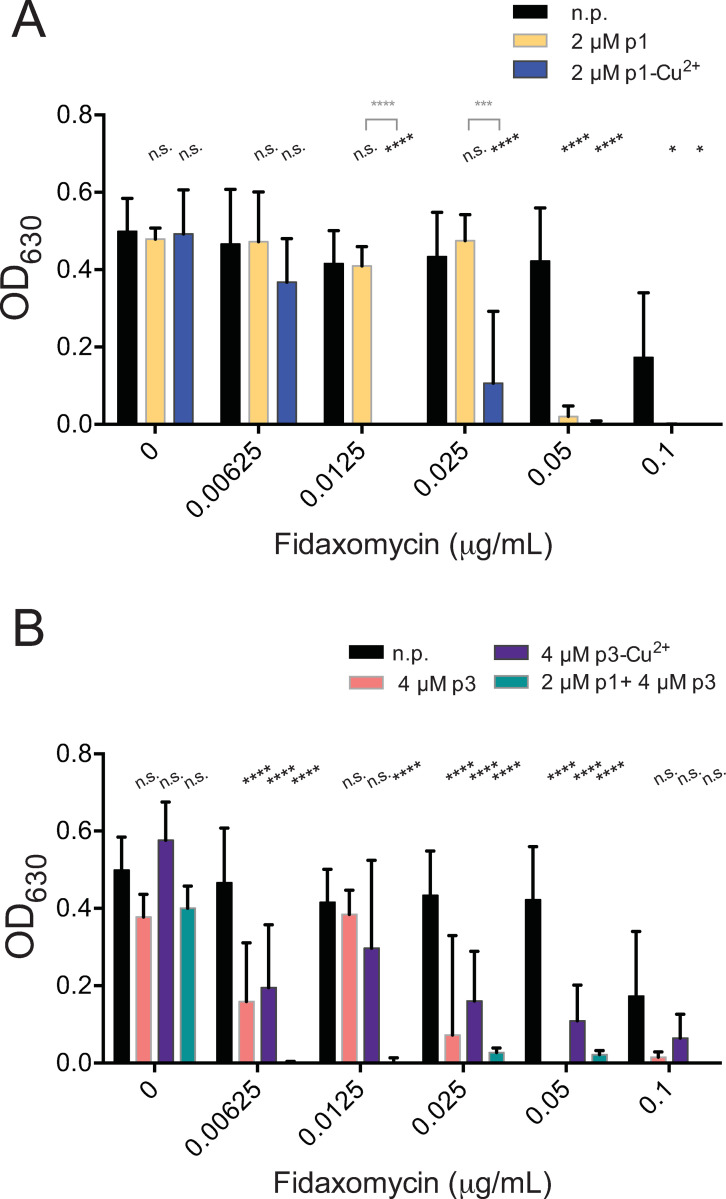
Piscidins synergize with fidaxomicin. Culture density after overnight growth in the presence of the indicated concentrations of fidaxomicin and sub-inhibitory concentrations of (A) p1 or p1-Cu^2+^complex or (B) p3 or p3-Cu^2+^complex. Data shown represent the means and standard deviations of at least three samples. Conditions are compared by two-way ANOVA. The comparison between samples containing fidaxomicin and those containing fidaxomicin and peptides is shown in black. When comparisons between the apo and holo forms of the peptides are statistically significant, they are shown in grey. n.p. no peptide, n.s. not significant, * *p* > 0.05, **** *p* > 0.0001.

We also tested fidaxomicin, an antibiotic which inhibits RNA polymerase at the time of transcription initiation; it thus has an intracellular target but does not bind to DNA. It is effective against *C*. *difficile* at much lower concentrations than metronidazole or vancomycin. The concentration necessary to suppress *C*. *difficile* growth, previously observed to be 0.256 μg/mL, was reduced 5-fold to 0.05 μg/mL by the presence of 2 μM p1 (Fig 3A and Table 1) [76]. The use of 2 μM p1-Cu^2+^ was even more effective, lowering the observed effective concentration 20-fold, to 0.0125 μg/mL (Fig 3A and Table 1). Using 4 μM p3 resulted in the same efficacy as 2 μM p1. However, even 0.1 μg/mL fidaxomicin, the highest concentration tested, did not inhibit growth below the 90% threshold in the presence of 4 μM p3-Cu^2+^ (Fig 3B and Table 1). While p3-Cu^2+^ did appear to enhance fidaxomicin efficacy at lower concentrations, it was less effective than the apo-state, thus the presence of copper ions did not appeared to boost the adjuvant activity of p3 against this antibiotic. Here, the mixture of 2 μM p1 and 4 μM p3 did display a significant adjuvant effect with the antibiotic, sensitizing *C*. *difficile* 40-fold, down to 0.00625 μg/mL, an enhancement far greater than that achieved by either peptide alone (Fig 3A and 3B and Table 1).

**Fig 4:**
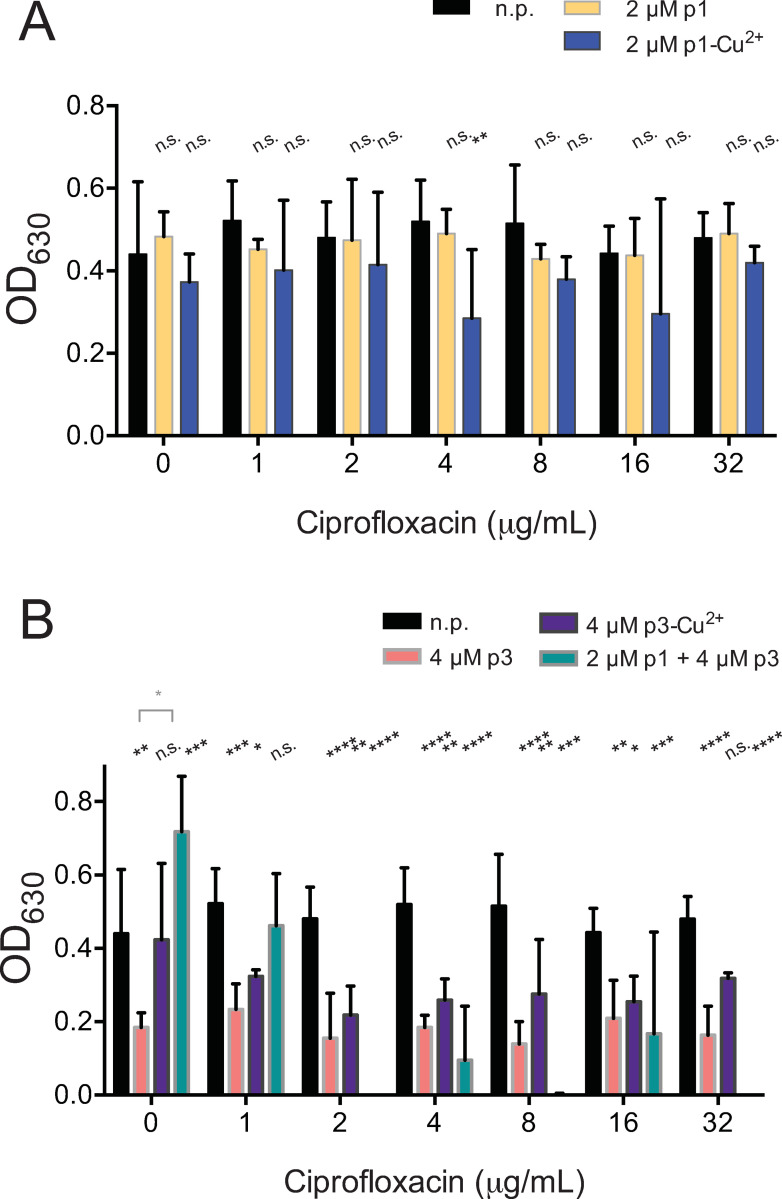
Piscidins do not synergize strongly with ciprofloxacin. Culture density after overnight growth in the presence of the indicated concentrations of ciprofloxacin and sub-inhibitory concentrations of (A) p1 or p1-Cu^2+^complex or (B) p3 or p3-Cu^2+^. Data shown represent the means and standard deviations of at least three samples. Conditions are compared by two-way ANOVA. The comparison between samples containing ciprofloxacin and those containing ciprofloxacin and peptides is shown in black. When comparisons between the apo and holo forms of the peptides are statistically significant, they are shown in grey.. n.p. no peptide, n.s. not significant, * *p* > 0.05, **** *p* > 0.0001.

We repeated these assays with ciprofloxacin, an antibiotic that targets topoisomerases to which *C*. *difficile* is highly resistant with a recorded MIC > 128 μg/mL. p1 alone had no discernible effect on clostridial growth in ciprofloxacin, while growth was suppressed 37% and 34% by 4 and 16 μg/ml antibiotic in the presence of the p1-Cu^2+^ complex (Fig 4A and Table 1). Interestingly, in all concentrations of ciprofloxacin tested, growth was suppressed 27%-67% by both the apo and holo forms of p3, and the presence copper had no apparent effect on this interaction. (Fig 4B and Table 1) The combination of p1 and p3 did appear to enable ciprofloxacin to suppress clostridial growth at 2, 8, and 32 μg/mL concentrations, with breakthrough growth observed at 4 and 16 μg/mL ciprofloxacin (Fig 4B).

### Piscidins activate antibiotics to enhance vegetative cell killing but do not affect dormant *C*. *difficile* spores

**Fig 5:**
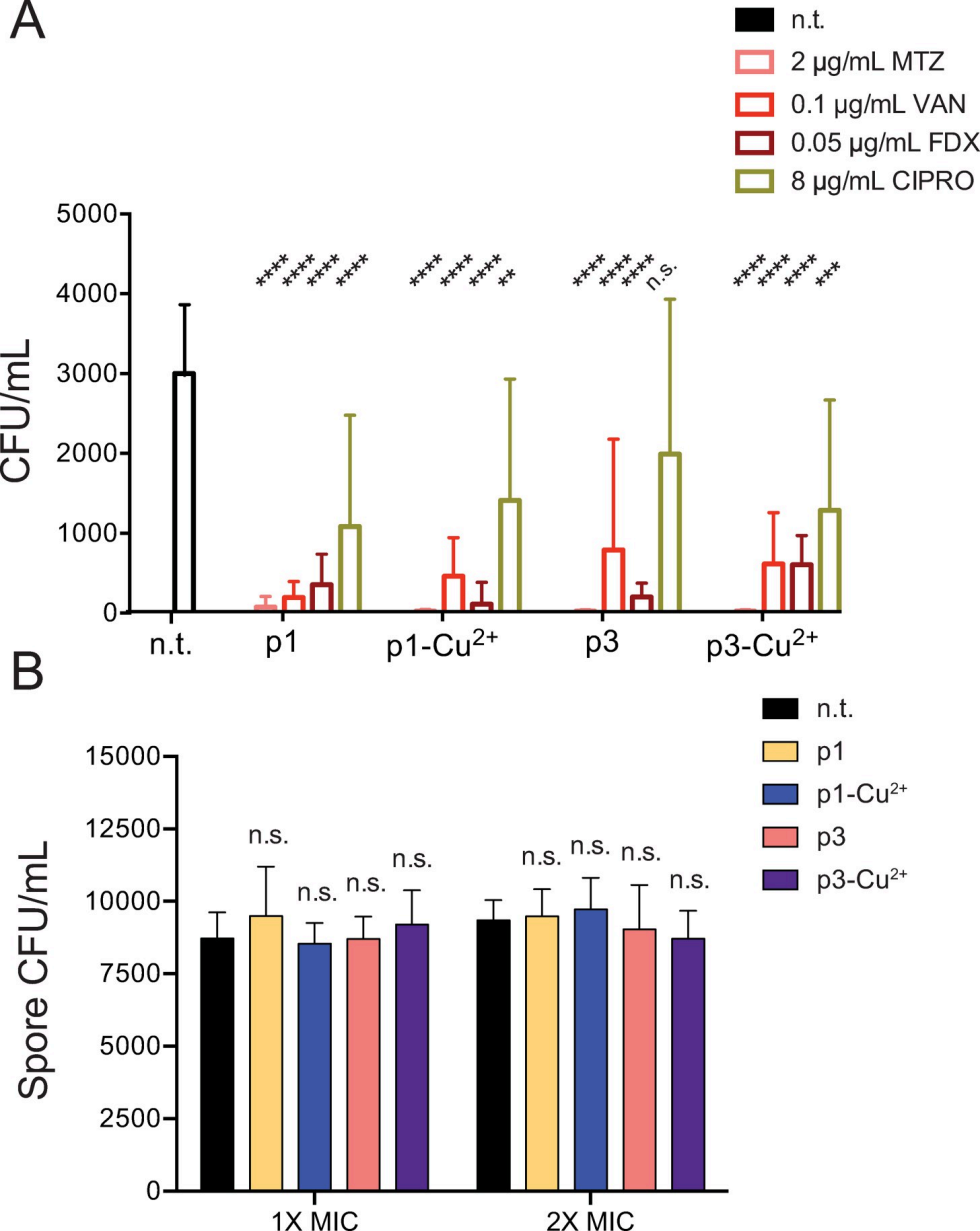
Piscidins reduce viability of vegetative cells but not dormant spores. (A) Combined treatment with sub-lethal concentrations of piscidins and effective antibiotics reduces viable cells, while piscidins exhibit much less bacteriocidal synergy with ciprofloxacin. Data shown represent the means and standard deviations of six samples. Treated samples are compared to the non-treated control by two-way ANOVA. n.t. not treated, n.s. not significant, ** *p* > 0.01, *** *p* > 0.001, **** *p* > 0.0001. (B) Dormant spores incubated with inhibitory concentrations of piscidins (4 μM p1, 8 μM p3) or in twice the inhibitory concentrations (8 μM p1, 15 μM p3) before exposure to germinant germinated at comparable rates to untreated spores. Data shown represent the means and standard deviations of at least six samples. Treated samples are compared to the non-treated control by two-way ANOVA. n.t. not treated, n.s. not significant.

To confirm that the combined piscidin-antibiotic treatments had killed the bacteria in the samples rather than temporarily suppressing their growth, the overnight exposures were repeated. After overnight incubation in conditions that contained the sub-lethal concentrations of piscidins i.e. 2 μM p1 (or p1-Cu^2+^) or 4 μM p3 (or p3-Cu^2+^) combined with antibiotic concentrations which inhibited overnight growth, samples were spread on BHIS plates and visible colonies were enumerated. While the number of culturable vegetative cells in the untreated controls was not very high after overnight growth, co-treatment with piscidins and sub-lethal concentrations of metronidazole, vancomycin, or fidaxomicin further reduced the number of viable cells in the samples (Fig 5A). Piscidins together with 8 μg/mL ciprofloxacin reduced the number of colonies roughly 2-fold, commensurate with the modest reduction seen in optical densities after ciprofloxacin treatment (Fig 5A). While liquid BHIS is not a growth medium that yields high numbers of spores, overnight incubation does result in the recovery of heat- and ethanol-resistant *C*. *difficile* spores [78]. Previously, cationic HDPs were not found to affect dormant spores of *Bacillus subtilis* due to lack of access to the plasma membrane [79,80]. Similarly, we found that p1 and p3, when present at or above the concentrations necessary to kill vegetative *C*. *difficile*, do not affect the viability of dormant *C*. *difficile* spores (Fig 5B).

### Piscidins increase ability of exogenous substances to penetrate *C*. *difficile* membranes

**Fig 6:**
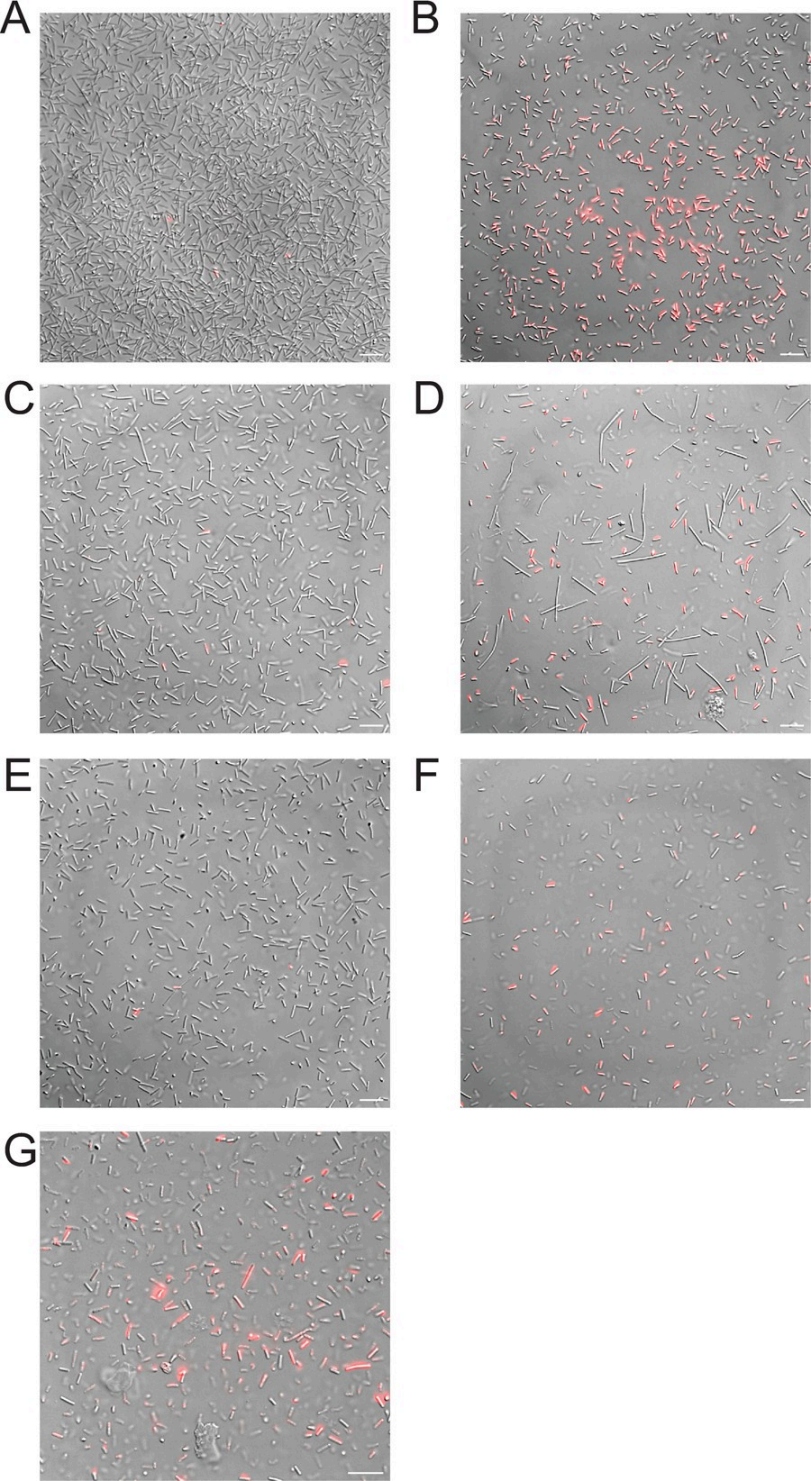
Piscidins increase uptake of exogenous substances by *C*. *difficile* cells. Representative images showing incorporation of membrane impermeable propidium iodide (PI) in (A) live untreated *C*. *difficile* cells and (B) *C*. *difficile* killed by oxygen exposure and ethanol. (C-F) PI uptake after anaerobic incubation with live *C*. *difficile* cells and inhibitory concentrations of (C) p1, (D) p1-Cu^2+^, (E) p3, (F) p3-Cu^2+^, or (G) p1 and p3 combined. Scale bars represent 5 μm in all images.

We have previously shown uptake of fluorescently labeled piscidins by live *C*. *difficile* cells, which appear to accumulate the labeled peptides at cell poles and division septa where the membranes exhibit curvature [53]. Here, we have assessed the ability of non-labeled piscidins to increase uptake of exogenous substances across the bacterial cell membrane. We treated live, exponentially growing *C*. *difficile* cells with sublethal peptide concentrations and the fluorophore propidium iodide (PI), which is not permeable across intact cell membranes and typically only stains dead or membrane-compromised cells [80]. Samples containing apo peptide were incubated for 30 minutes before unincorporated dye was washed away and samples were placed in sealed rose chambers for live cell microscopy [53,81]. Samples containing metalated peptides were incubated for 10 minutes, as the peptide-copper complexes began to stimulate visible cell lysis within 30 minutes of exposure. PI uptake was extremely low in live C. difficile in anaerobic conditions (Fig 6A) and was much greater in cells exposed to cytotoxic levels of oxygen and ethanol (Fig 6B). The apo forms of p1 and p3 did not visibly increase PI uptake (Fig 6C and 6E), but the metalated forms of both peptides did increase the number of red (dead) cells present in the fields of view examines (Fig 6D and 6F). The combination of sublethal levels of p1 and p3 also visibly increased PI uptake, resulting in red fluorescent cells (Fig 6G). While qualitative, these results illustrate increased penetration of exogenous membrane-impermeable substances in the presence of piscidins.

### Piscidins permeabilize *C*. *difficile* cell membranes

**Fig 7:**
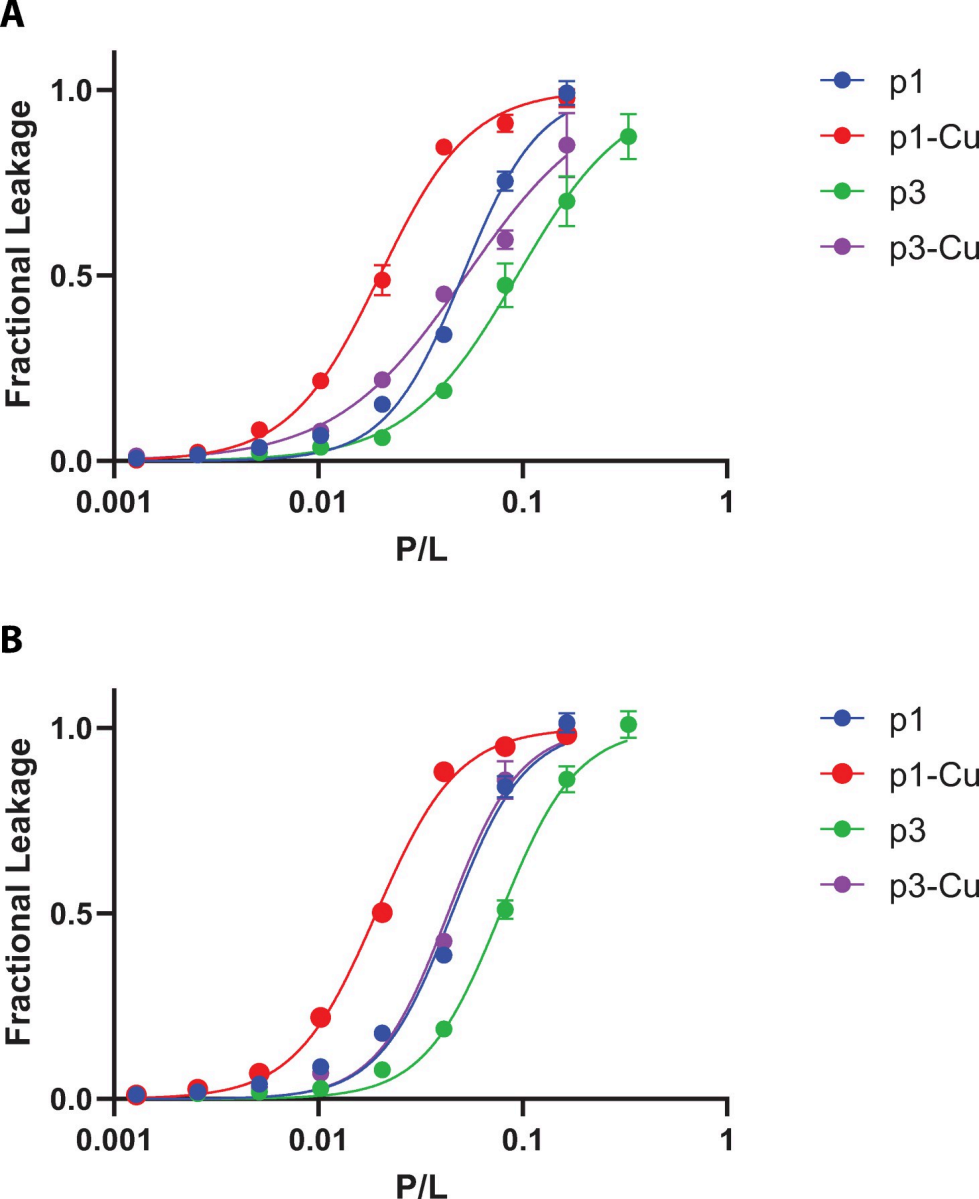
Piscidins cause leakage of glycolipid containing membranes. Fractional leakage of calcein through membrane vesicles comprised of 45 μM suspensions of 2:1 (16:0 18:1) POPG: (16:0–18:1) DG-Glucose is shown as a function of the peptide/lipid ratio at (A) 37°C and (B) 45°C. The assay was duplicated and one representative replicate is shown. Each data point is the average of independent measurements made in triplicate. The EC_50_ values based on fitting the two duplicates are provided in Table 2.

To qualitatively assess the activity of the piscidin peptides at *C*. *difficile* membranes, we quantified their ability to release calcein entrapped in vesicles constituted of lipid mimicking the bilayer of the pathogen. We used lipids representative of unique membrane composition of *C*. *difficile* [82–84]. Importantly, *C*. *difficile* lacks phosphatidylethanolamine (PE), a phospholipid commonly present in bacterial membranes, but contains glycolipids [82,83]. Here, we used a mixture of POPG and DG-glucose to model the membrane of *C*. *difficile*. The POPG component mimics the high anionic content of its membrane while DG-glucose recapitulates the presence of zwitterionic glycolipids [82]. Normalized fluorescence intensity measurements were plotted against the peptide-to lipid ratios and their sigmoidal shape is indicative of a cooperative mechanism of action (Fig 7) [85]. This is consistent with leakage assays previously reported involving these peptides in other lipid systems [42]. The EC_50_ values are shown in Table 2, where higher EC_50_ value correspond to enhanced membrane activity of the peptides. In the case of both p1 and p3, the holo states achieved higher permeability compared to the apo forms by a factor of ∼2–3, consistent with previous reports (Fig 7) [57,58,62]. p1-Cu^2+^ exhibited the highest activity with an EC_50_ of 1:52 while this value was 1:20 for P3-Cu^2+^. We previously demonstrated that the stronger membrane activity of p1 compared to p3 correlates with its enhanced ability to be retained by the bilayer, and thus can form longer-lived defects [57]. The metalated state of each peptide is more active than its apo-form due to enhanced depth of insertion. To the best of our knowledge, the phase transition temperature (T_m_) of DG-glucose and similar glycolipids has not been reported but glycolipids tend to increase T_m_ due to changes in packing of molecules in the membrane [86]. Since the melting point could be in the range of 37°C [86], we tested the higher temperature of 45°C. However, similar EC_50_ values were obtained at both temperatures. Together with the fact that the EC_50_ obtained are similar to those reported previously for p1 and p3 with plain phospholipids used above their T_m_ [57,67,77,87], we conclude that the phase transition was already reached at 37°C.

**Table 2:** EC_50_ values (as peptide-to-lipid (P/L) ratios) for piscidin peptides acting on *C*. *difficile* model membranes containing a glycolipid*.

EC_50_ (P/L)	37°C	45°C
p1	1:19	1:19
p1-Cu^2+^	1:52	1:50
p3	1:12	1:12
p3-Cu^2+^	1:20	1:20

*In terms of the values bracketing the 95% confidence interval, the error is ± 2 on the L part of the P/L ratio.

**Fig 8:**
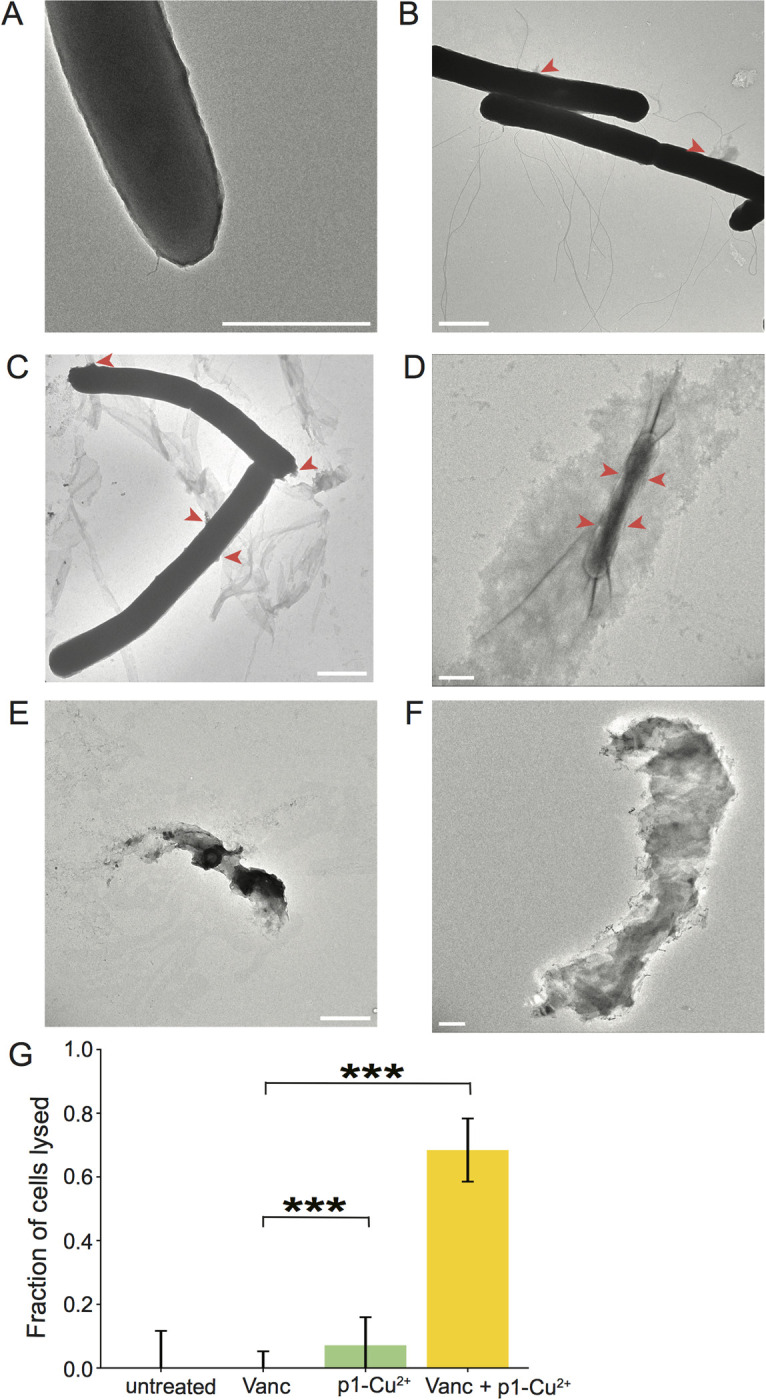
Piscidin and pisicidin-vancomycin co-treatment damage the cell envelope allowing cytoplasmic leakage. Representative TEM images of (A) untreated *C*. *difficile* (B) *C*. *difficile* treated with vancomycin (C, E) *C*. *difficile* treated with p1-Cu^2+^ (D, F) *C*. *difficile* treated with p1-Cu^2+^ and vancomycin. Scale bars represent 1 μm in each panel. (G) Statistical analysis of the cells that were lysed after treatment with vancomycin, p1-Cu^2+^, or vancomycin + p1-Cu^2+^. *** indicates p<0.0002 based on a proportion z-test.

To directly visualize the effects of piscidin treatment, antibiotic stress, and piscidin-antibiotic co-treatment on the *C*. *difficile* cell envelope, we fixed cells after brief treatment and visualized their surface integrity by transmission electron microscopy. We exposed cells to 4 μM p1-Cu^2+^, the most membrane-active peptide, and 0.5 μg/mL vancomycin, the cell-wall active antibiotic, as well as a combination of the two. Exponentially growing cells were incubated with peptide and/or antibiotic for five minutes before fixation, as there was a visible loss of cell density in the co-treated samples after longer treatments (data not shown). Seven untreated cells were observed, of which none had any visible morphological damage (Fig 8A). Of the 22 cells treated with vancomycin alone, 8 showed signs of cytoplasm leakage due to cell wall damage (Fig 8B). Among 14 cells treated with p1-Cu^2+^, 8 showed sites of membrane leakage (Fig 8C). Among 19 cells co-treated with p1-Cu^2+^ and vancomycin, 13 exhibited severe to complete cell lysis, including hollow cell envelopes that appeared to have lost much of their cytoplasmic content within five minutes of treatment (Fig 8D). Notably, these completely lysed cells, were not observed in the untreated or vancomycin-treated conditions (Fig 8E and 8F), and only one such completely lysed cell was seen in the cells treated p1-Cu^2+^ only. A statistical analysis of the proportion of lysed cells in the dual treated condition (13 of 19, or proportion p = 0.68) compared to lysed cells in the vancomycin alone condition (0 of 22, or proportion p = 0) or the p1-Cu^2+^ (1 of 14, proportion of p = 0.07) finds that this difference is highly statistically significant (P < .0001 for Dual treated vs vancomycin, and P < .0002 for dual treated versus p1-Cu^2+^ alone). Thus, the EM imaging confirms the finding that the dual treatment is synergistic, and far more effective than either vancomycin or p1-Cu^2+^ alone.

## Discussion

We have determined that co-treatment with piscidin HDPs enhances the efficacy of clinically relevant antibiotics used to treat CDI. The piscidins exhibit synergistic effects with antibiotics that have a wide range of cellular targets, suggesting that they non-specifically permeabilize the cell membrane and allow increased uptake of antibiotic compounds. However, the degree of interaction varied between the peptide/drug combinations. Vancomycin was potentiated most strongly by p1-Cu^2+^, followed by p3-Cu^2+^, and was affected much less by the apo form of either peptide. Vancomycin affects cell wall biosynthesis in Gram positive bacteria, so it appears likely that a simultaneous assault upon the cell envelope by vancomycin and membrane-active p1-Cu^2+^, the peptide with the greatest effect on membrane permeability, creates an effect greater than the sum of its parts. This was directly visualized by transmission electron microscopy of the clostridial cell surface. Cells treated with a sub-inhibitory concentration of 0.5 μg/mL vancomycin for only five minutes showed envelope damage and cytoplasm leakage, but this treatment was insufficient to cause cell lysis. Similarly, a five minute exposure to sub-inhibitory 4 μM pi-Cu^2+^ caused envelope damage but no widespread lysis. However, the combination of the two sub-inhibitory treatments was lethal to *C*. *difficile*, completely lysing the majority of cells examined. The presence of the peptide substantially enhanced the antibiotic efficacy, effectively sensitizing *C*. *difficile* to trace amounts of vancomycin to which it would otherwise be resistant. Vancomycin resistance in *C*. *difficile* can be acquired from plasmids encoding putative peptidoglycan modification genes or can arise from mutations in the chromosomal *vanG*_*Cd*_ operon which encodes peptidoglycan processing enzymes [88–90]. Physical perturbation of the plasma membrane and/or DNA damage caused by piscidins are unlikely to provide selective pressure for alterations in or acquisition of peptidoglycan processing genes, so it is unlikely that co-application with piscidins would stimulate the development of vancomycin resistance.

Another strong enhancing effect was observed between p3 and metronidazole, both of which target DNA. Interestingly, the presence of copper ions appeared detrimental to the metronidazole-piscidin interaction, as both p1 and p3 were more effective in their apo forms. While some clinical cases of metronidazole-resistant CDI have been attributed to a plasmid encoding a 5-nitroimidazole reductase gene, clostridial metronidazole resistance does not appear to depend on mutation of any one gene but to involve decentralized reduction of metronidazole by multiple endogenous reductases using heme scavenged from the growth medium or host as an electron donor [91–94]. It is possible that the presence of copper, a redox stressor for cells in its own right, causes metal-bound piscidins to stimulate such reductase activity more strongly than the apo peptides.

There are intuitive potential interactions between p1 and vancomycin at the cell envelope, and between p3 and metronidazole in the presence of DNA. Fidaxomicin, which targets RNA polymerase, has no such shared target with either piscidin. Fidaxomicin, active against *C*. *difficile* at much lower concentrations than either vancomycin or metronidazole, was effective at even lower concentrations in the presence of either p1 or p3. Here copper strongly enhanced the antibiotic-p1 interaction, while appearing detrimental to the antibiotic-p3 interaction. It is notable that copper strongly enhanced piscidin-dependent membrane leakage for both peptides, suggesting that the metal ions increase either the size or the duration of the p1-mediated membrane perturbations that facilitate antibiotic entry into the bacterial cell. This could facilitate fidaxomicin entry into the cell in the presence of p1-Cu^2+^ even if copper ions are somehow inhibitory of fidaxomicin activity in the presence of p3-Cu^2+^.

The activity of ciprofloxacin in the presence of combined peptides was the most surprising result of these investigations (Fig 5A). Concentrations of this antibiotic that had no effect on clostridial growth on their own did partially suppress *C*. *difficile* replication in the presence of p1 and p3, with p3 being more effective than p1. Copper appeared to have no effect on phenomenon. Clostridial resistance to ciprofloxacin and other fluoroquinoline antibiotics is due to amino acid substitutions in its DNA gyrase A and B subunits [95,96]. It is possible that p3-mediated DNA damage affects topoisomerase function in a manner that resensitizes cells to ciprofloxacin. Future comparisons of p3 activity against fluoroquinoline-sensitive and fluoroquinoline-resistant bacterial strains will be necessary to confirm this possibility. If true, it suggests that p3-derived treatments could someday be administered with fluoroquinolones as adjuvants to reduce the risk of antibiotic-induced CDI.

Administering p1 and p3 together enhanced fidaxomicin and ciprofloxacin activity more strongly than either peptide alone, in either their apo or holo form. However, the interactions of the peptide combination with vancomycin and metronidazole were less straightforward. The p1-p3 mixture potentiated vancomycin more strongly than p1, p3 or p3-Cu^2+^ but its interaction with vancomycin was not significantly different from that of p1-Cu^2+^. Similarly, the p1-p3 mixture potentiated metronidazole more strongly than p1, p1-Cu^2+^, or p3-Cu^2+^, but had no significant difference from p3. The vancomycin-p1-Cu^2+^ and metronidazole-p3 combinations are the ones that appear to exhibit maximal mutual enhancement and for which we are able to propose a specific mechanism based on overlapping targets. It appears the mixture of peptides with their complementary mechanisms of action is able to interact with multiple antibiotics to recapitulate the performance of individual peptides in their optimal combinations.

Overall, these results indicate that while piscidins are effective adjuvants to clinically relevant antibiotics, reducing the concentrations necessary to suppress clostridial growth in every condition tested, their efficacy can be optimized by specifically using knowledge from mechanistic studies to design HDP-drug combinations. Our ciprofloxacin studies suggest that antibiotics which have lost efficacy could perhaps be ‘rescued’ by combinatorial treatment with antimicrobial peptides. Such combinations could also increase the lethality to bacteria of currently approved dosages of effective antibiotics, reducing the risk of incomplete treatment leading to bacterial antibiotic resistance.

## Supporting information

S1 File Piscidin vancomycin data. (CSV)

S2 File Piscidin metronidazole data. (CSV)

S3 File Piscidin fidaxomicin data. (CSV)

S4 File Piscidin cipro data. (CSV)

S5 File Piscidin vegetative CFU data. (CSV)

S6 File Spore data. (CSV)

S7 File Raw data for Fig 7A. (CSV)

S8 File Raw data for Fig 7B. (CSV)

S9 File TEM quantifications. (CSV)
